# Site-specific effects of 800- and 850-nm forehead transcranial photobiomodulation on prefrontal bilateral connectivity and unilateral coupling in young adults

**DOI:** 10.1117/1.NPh.10.2.025012

**Published:** 2023-06-05

**Authors:** Sadra Shahdadian, Xinlong Wang, Shu Kang, Caroline Carter, Hanli Liu

**Affiliations:** University of Texas at Arlington, Department of Bioengineering, Arlington, Texas, United States

**Keywords:** broadband near-infrared spectroscopy, transcranial photobiomodulation, infra-slow oscillation, prefrontal cortex, cytochrome c oxidase, functional connectivity

## Abstract

**Significance:**

Transcranial photobiomodulation (tPBM) is a noninvasive neuromodulation method that facilitates the improvement of human cognition. However, limited information is available in the literature on the wavelength- and site-specific effects of prefrontal tPBM. Moreover, 2-channel broadband near-infrared spectroscopy (2-bbNIRS) is a new approach for quantifying infra-slow oscillations (ISO; 0.005 to 0.2 Hz) of neurophysiological networks in the resting human brain *in vivo*.

**Aim:**

We aim to prove the hypothesis that the hemodynamic and metabolic activities of the resting prefrontal cortex are significantly modulated by tPBM and that the modulation is wavelength- and site-specific in different ISO bands.

**Approach:**

Noninvasive 8-min tPBM with an 800- or 850-nm laser or sham was delivered to either side of the forehead of 26 healthy young adults. A 2-bbNIRS unit was used to record prefrontal ISO activity 7 min before and after tPBM/sham. The measured time series were analyzed in the frequency domain to determine the coherence of hemodynamic and metabolic activities at each of the three ISO frequency bands. Sham-controlled coherence values represent tPBM-induced effects on neurophysiological networks.

**Results:**

Prefrontal tPBM by either wavelength and on either lateral side of the forehead (1) increased ipsilateral metabolic-hemodynamic coupling in the endogenic band and (2) desynchronized bilateral activity of metabolism in the neurogenic band and vascular smooth-muscle hemodynamics in the myogenic band. Site-specific effects of laser tPBM were also observed with significant enhancement of bilateral hemodynamic and metabolic connectivity by the right prefrontal 800-nm tPBM.

**Conclusions:**

Prefrontal tPBM can significantly modulate neurophysiological networks bilaterally and coupling unilaterally in the human prefrontal cortex. Such modulation effects are site- and wavelength-specific for each ISO band.

## 1 Introduction

### 1.1 Infra-slow Oscillations in the Resting Human Brain

The central nervous system, particularly the brain, is one of the dominant consumers of oxygen and glucose in the human body due to its high levels of metabolism, even at rest.^1^^,^^2^ Neural oxidative respiration, which is closely related to oxygen supply level, modulates energy metabolism in neurons.^3^^,^^4^ Previous studies have demonstrated the role of intrinsic rhythmic relaxation-contraction (i.e., vasomotion) of vascular walls in human cerebral metabolic and hemodynamic activity.^5^^–^^9^ Vasomotion consists of spontaneous infra-slow oscillations (ISO) in the frequency range of 0.005 and 0.2 Hz,^10^^,^^11^ which are related to different human brain functions.^12^ In addition, disturbances in vasomotion have been associated with aging, neurological disorders, and other diseases such as atherosclerosis,^13^ cardiovascular disease,^14^ and Alzheimer’s disease.^15^ In other studies published by our group, we have shown that vasomotion and its frequency-domain characteristics over the prefrontal cortex at rest can be quantified as brain activity features in healthy humans.^16^^,^^17^

**Fig. 1:**
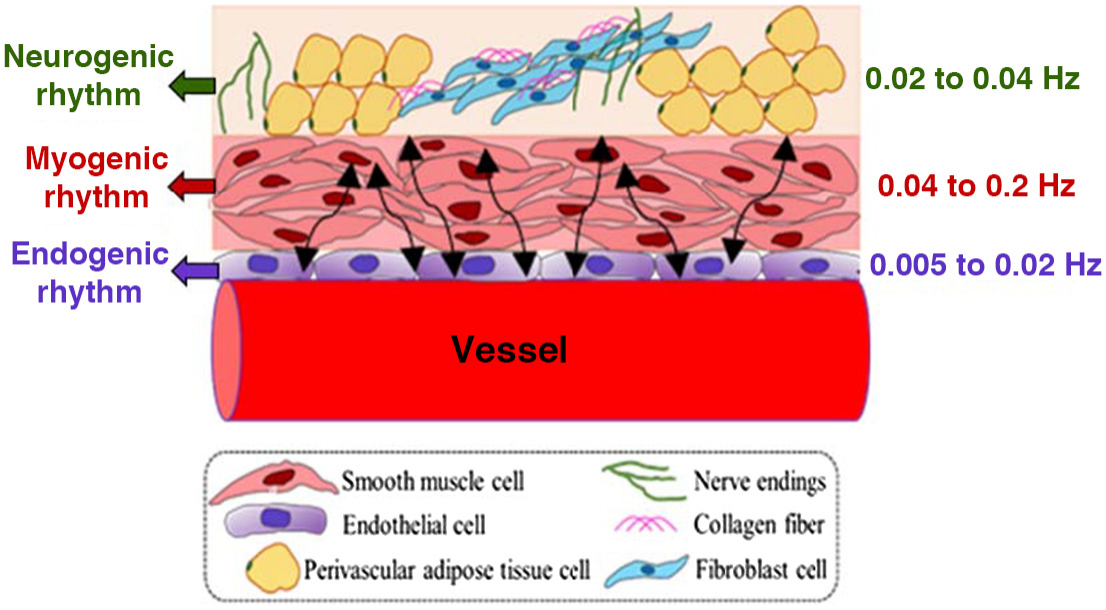
A schematic illustration of a piece of blood vessel surrounded with three anatomical components^25^ that facilitate spontaneous ISO with endogenic (0.005 to 0.02 Hz), neurogenic (0.02 to 0.04 Hz), and myogenic rhythms (0.04 to 0.2 Hz).

In particular, the infra-slow vasomotion is independent of respiration and heartbeat^6^^,^^18^^–^^20^ and consists of three distinct physiologically/biochemically-sourced components.^21^ Briefly, oscillations in the release of potent vasoactive factors lead to rhythmic physiological activities in the endogenic frequency band (E; 0.005 to 0.02 Hz), which corresponds to relaxation-contraction cycles in the endothelial layer of the vascular wall. The neurogenic component (N; 0.02 to 0.04 Hz), on the other hand, is sourced from vasoactive ions and neurotransmitters released from neurons. The myogenic activity (M, 0.04 to 0.2 Hz) represents the dilation-contraction of smooth muscle cells on the vessel wall.^22^^–^^24^ Figure 1 schematically illustrates anatomical structures and respective rhythm frequencies for these three ISO components.^25^

### 1.2 Broadband Near-infrared Spectroscopy of the Human Brain *In Vivo*

Functional near-infrared spectroscopy (fNIRS),^12^ functional magnetic resonance imaging,^26^ and transcranial cerebral Doppler^27^ are common methods for detecting haemodynamic ISO. Nevertheless, a better understanding of the mechanism underlying human cerebral metabolism requires concurrent measurements of haemodynamic and metabolic activities in the region of interest. Alternatively, mitochondrial activity and oscillations can be quantified as a direct method for monitoring metabolic ISO.^28^ In this method, the metabolic state of living tissues can be detected by the concentration of redox-state cytochrome c oxidase ([CCO]), which is the terminal enzyme in the mitochondrial respiratory chain.^29^^–^^32^

Previous studies have demonstrated the capability of broadband near-infrared spectroscopy (bbNIRS) to quantify changes in the concentration of oxygenated haemoglobin (Δ[HbO]), deoxygenated haemoglobin (Δ[HHb]), and redox-state [CCO] (Δ[CCO]).^33^^,^^34^ The underlying principle of this method is based on the absorption and scattering characteristics of these chromophores in living tissue.^33^^,^^35^^–^^38^ Because of the lower concentration of [CCO] in tissue as compared to [HbO] and [HHb], multiple wavelengths are required in the near-infrared spectroscopy (NIRS) system to accurately determine the changes in the three chromophores in the living tissue.^39^^–^^41^

### 1.3 Neurophysiological Effects of Transcrancial Photobiomodulation by Near-Infrared Light

Transcranial photobiomodulation (tPBM), a noninvasive method of neuromodulation,^42^^,^^43^ has been shown to alter cerebral hemodynamic and metabolic states.^34^^,^^44^^,^^45^ This method uses a low-intensity laser or light-emitting diode to deliver near-infrared light to the human head. It has been shown that a portion of the delivered light reaches the human cortex and facilitates improvement in cognitive function in people with or without neurological disorders.^42^^,^^43^^,^^46^ Previous studies have demonstrated the dose-dependent nature of tPBM in the modulation of Δ[HbO] and Δ[CCO], especially in the human prefrontal cortex.^34^^,^^44^^,^^45^ Furthermore, 1064-nm tPBM delivered to the right human forehead has been proven to alter cerebral hemodynamic, metabolic, electroencephalogram (EEG) power, and electrophysiological functional connectivity at rest.^34^^,^^45^^,^^47^^,^^48^

Our group recently introduced a set of hemodynamic and metabolic characteristics quantified by frequency-domain connectivity analysis of hemodynamic and metabolic ISO activity in the prefrontal cortex, as assessed by dual-channel bbNIRS measurements.^16^^,^^17^ These metrics are (1) bilateral hemodynamic (i.e., Δ[HbO]) connectivity (${\mathrm{bCON}}_{\mathrm{HbO}}$), (2) bilateral metabolic (i.e., Δ[CCO]) connectivity (${\mathrm{bCON}}_{\mathrm{CCO}}$), (3) unilateral hemodynamic-metabolic coupling (uCOP) on the left, and (4) right side of the prefrontal cortex. In addition, we demonstrated that these constant and relatively reproducible metrics can be considered potential features of the human prefrontal cortex in young healthy adults.^17^ We have also shown distinct alterations in these metrics across all three frequency bands in response to 1064-nm tPBM.^16^

However, very limited information is available in the literature on the wavelength- and site-specific effects of prefrontal tPBM. While Pruitt et al.^49^ offered preliminary results on wavelength-dependent alterations in Δ[HbO] and Δ[CCO] of the human forearm with three wavelengths, more sham-controlled neurophysiological measurements are needed to better understand the underlying effects of tPBM on behavioral (or neuropsychological) and neurophysiological alterations that would help the optimal selection of tPBM stimulation parameters and setting conditions.

### 1.4 Aims and Outline of this Study

This study aimed to prove the hypothesis that hemodynamic and metabolic activities in the ISO of the resting human forehead are significantly modulated by tPBM stimulation conditions and that the modulation is wavelength- and site-specific, as well as distinct in different ISO bands. Specifically, a 2-channel bbNIRS (2-bbNIRS) system was used to acquire the cerebral Δ[HbO] and Δ[CCO] time series from the forehead of young healthy human participants before and after an 8-min tPBM/sham. Five different 8-min stimulation settings were given to each participant: (1) right forehead 800-nm laser, (2) right forehead 850-nm laser, (3) right forehead sham, (4) left forehead 800-nm laser, and (5) left forehead sham. The measured time series were then analyzed to determine the coherence of the hemodynamic and metabolic ISO over the three frequency bands (i.e., E/N/M). Finally, four physiological metrics were derived to characterize the connectivity/coupling between each pair of signals in response to the respective tPBM conditions.

## 2 Materials and Methods

### 2.1 Participants

A total of 31 healthy human participants were recruited from the local community of the University of Texas at Arlington. The patients were screened using the same inclusion criteria as those used by Wang et al.^50^^,^^51^ Since the bbNIRS system was highly sensitive to motion artifacts, five subjects with excessive motion during the experiment were excluded from the analyzed data. After exclusion, a total of 26 young healthy humans (14 males and 12 females, mean ± SD age=22.4±2.3 years) participated in 5 visits separated by at least 7 days to minimize post-tPBM residual effects. Each participant was measured under five specific conditions: (1) right prefrontal 800-nm tPBM (R800), (2) right prefrontal 850-nm tPBM (R850), (3) right prefrontal sham (RS), (4) left prefrontal 800-nm tPBM (L800), and (5) left prefrontal sham (LS) stimulation. Sequences of the five experiments were randomly assigned to each subject. The data obtained under the two sham conditions (RS and LS) were previously analyzed in our recent study on bilateral hemodynamic/metabolic connectivity and unilateral hemodynamic-metabolic coupling in the resting human brain.^17^ The Institutional Review Board of the University of Texas at Arlington approved all experimental procedures. All measurements were conducted with informed consent from each participant.

### 2.2 Experiment Setup and Protocol

**Fig. 2:**
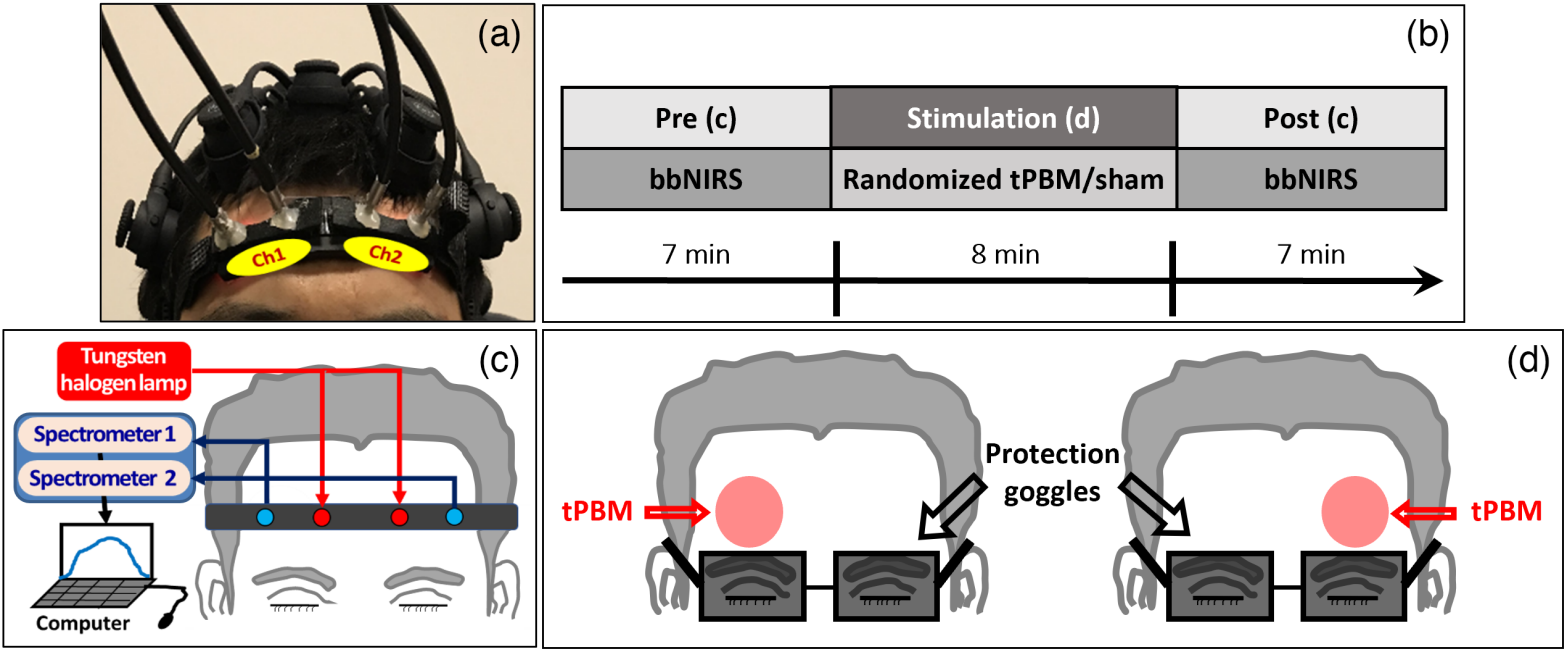
(a) Experiment setup including two channels of bbNIRS on two lateral sides of the forehead and EEG cap. The electrophysiological data collected by 2-bbNIRS were the focus in this study. (b) The protocol of this study consisted of 5 visits with a 7-min pre-stimulation, an 8-min randomized tPBM or sham, and 7-min post-stimulation period. The bbNIRS data were collected concurrently during pre- and post-stimulation. (c) Illustration of the light source, two spectrometers, 2-channel probe bundle for 2-bbNIRS measurements. (d) Illustration of the delivery location for the right and left tPBM/sham.

The original study employed a dual-mode experimental setup consisting of a 2-bbNIRS system and a 19-channel EEG. However, in this study, we focused on a dataset obtained using only 2-bbNIRS. As described in Ref. 17, 2-bbNIRS was implemented to measure NIR spectral changes on the bilateral foreheads of the participants under pre-tPBM/sham stimulation (resting-state) and post-tPBM/sham stimulations. Δ[HbO] and Δ[CCO] were quantified based on the absorption and scattering coefficients of the major chromophores in the tissue. The experimental setup of the 2-bbNIRS probes and EEG cap is shown in Fig. 2(a), and the experimental protocol for tPBM and sham experiments is illustrated in Fig. 2(b). The total experiment time was 22 min, including a 7-min pre-stimulation (rest), an 8-min randomized tPBM/sham, followed by a 7-min post-stimulation period. Specifically, two separate bbNIRS channels, each with a 3-cm source-detector separation, were placed on the subject’s forehead before and after tPBM/sham delivery to either lateral side of the forehead^17^ [also see Fig. 2(c)]. Furthermore, as illustrated in Fig. 2(d), the 2-bbNIRS holder was removed during the 8-min stimulation period. This figure shows the stimulation sites for the right and left tPBM/sham delivery. In the case of right-lateral stimulation, the right channel [Ch1 in Fig. 2(a)] is called the ipsilateral channel, and the left channel [Ch2 in Fig. 2(a)] is called the contralateral channel. This terminology is followed for the left-lateral stimulation. This setup enabled us to simultaneously measure the optical spectral alterations on both the ipsilateral and contralateral sides with respect to the stimulation site.

Dosages for tPBM used in this study were the same as those previously published.^49^ The peak irradiances at the center of the 4.1 cm diameter laser beams were 310 and $330\;\mathrm{mW}/{\mathrm{cm}}^{2}$ for 800- and 850-nm lasers, respectively. These two lasers were not fully collimated and had the approximate irradiances of 190 and $210\;\mathrm{mW}/{\mathrm{cm}}^{2}$ on 1 cm peripheral regions of the beams. Sham stimulation was delivered with the laser device turned on at a low power of 0.1 W for 8 min, whereas the laser aperture was also covered by a black cap. A power meter was used to confirm that the actual output power of the laser in the presence of the cap was zero.

The participants were asked to sit comfortably on a sofa chair. They were also asked to close their eyes during the entire experiment, without falling asleep. For eye protection, the participants and experimenters wore a pair of laser-protection goggles during both tPBM and sham stimulation.

### 2.3 Data Analysis

As reported in our previous study, the hemodynamic and metabolic characteristics (obtained from the pre-stimulation period) of the human prefrontal cortex at rest were relatively constant among healthy humans.^17^ Alterations in Δ[HbO] and Δ[CCO] metrics after 8-min of tPBM would represent the physiological effects of tPBM.

**Fig. 3:**
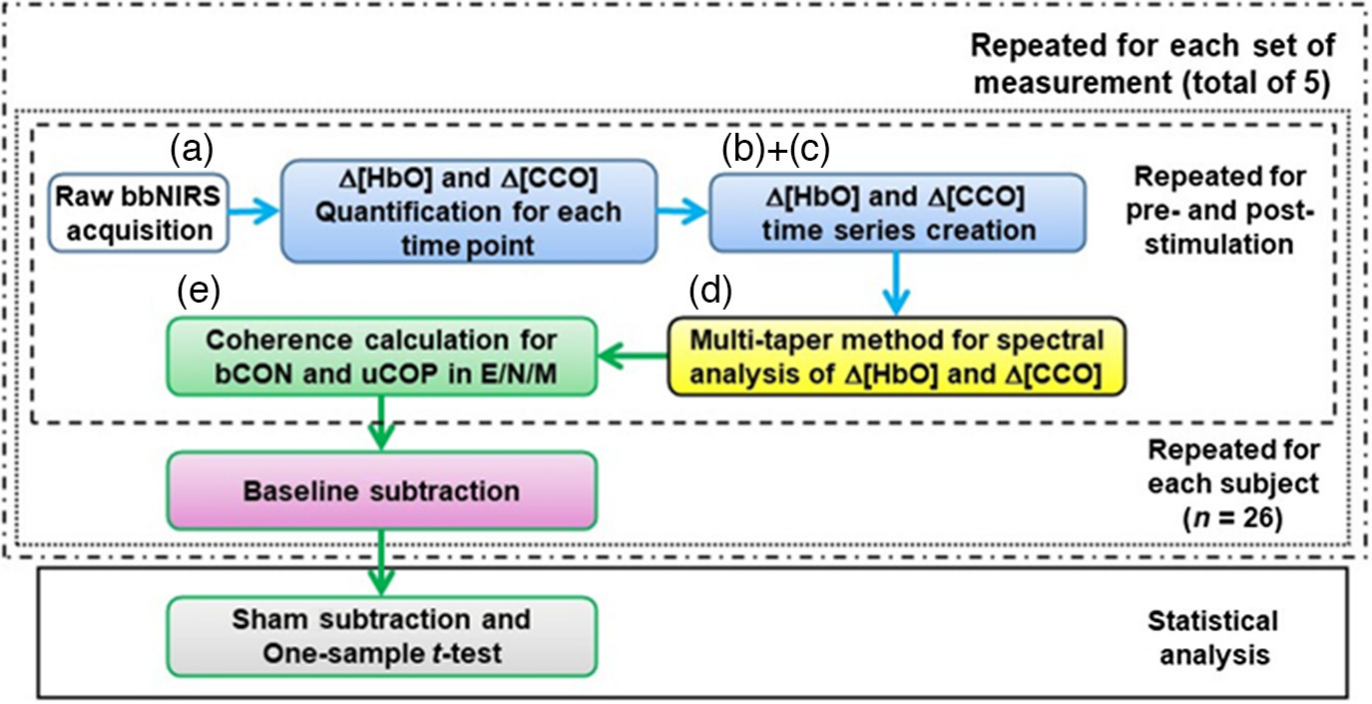
The data processing flowchart with five steps. Step 1: Δ[HbO] and Δ[CCO] quantification at each time point and construction of time series (blue boxes); Step 2: amplitude and phase decomposition using the multi-taper method (yellow box); Step 3: determination of four types of coherences for each E/N/M bands (green box). Steps 1 to 3 were repeated for pre- and post-stimulation periods (outlined by the dashed box). Step 4: baseline subtraction for coherence values (purple box). This step was repeated for each of the 26 participants (outlined by the second middle dotted box) and then for each of the five measurements (outlined by the outer dash-dotted box). Step 5 marks a two-step statistical analysis (gray box and solid box): sham subtraction followed by one-sample t-tests used to identify group-level, sham-controlled changes for respective coherence parameters in response to tPBM. Note that labels of (a)–(e) correspond to the data shown in Fig. 4 in the Results section.

In this study, we performed frequency-domain analyses; five steps were taken to quantify prefrontal hemodynamic and metabolic ISO activity, as outlined in Fig. 3. As Step 1 (blue boxes in Fig. 3), the Δ[HbO] and Δ[CCO] time series were constructed after converting the raw spectral data to Δ[HbO] and Δ[CCO] at each time point. The theoretical knowledge and calculation algorithms are detailed in the Supplementary Material and shown in Fig. S1 for general readers. In Step 2 (yellow box in Fig. 3), the frequency-dependent amplitude and phase of the time series were decomposed using the multitaper method (MTM) for coherence analyses. Step 3 was to estimate the coherence between each pair of the four time series among $\Delta {\left[\mathrm{HbO}\right]}_{\text{right}}$, $\Delta [\mathrm{HbO}{]}_{\text{left}}$, $\Delta {\left[\mathrm{CCO}\right]}_{\text{right}}$, and $\Delta {\left[\mathrm{CCO}\right]}_{\text{left}}$ (green box in Fig. 3). Similar to the previous approach,^17^ four physiologically interpretable pairs of these signals were formed: ${\mathrm{bCON}}_{\text{HbO}}$, ${\mathrm{bCON}}_{\text{CCO}}$, and unilateral hemodynamic-metabolic coupling on the ipsilateral (${\mathrm{uCO}}_{\text{Ipsi}}$) or contralateral (${\mathrm{uCOP}}_{\text{Contra}}$) side to the tPBM/sham site. In Step 4, the abovementioned three steps were performed for both the pre- and post-stimulation periods separately, and the obtained coherence values were the baseline (or pre-stimulation) subtracted as the fourth step (purple box in Fig. 3). This step was performed to assess the effect of sham/tPBM more efficiently (after baseline removal) and was repeated for each of the 26 subjects (dotted box in Fig. 3) for each set of five measurements (dot-dash box in Fig. 3). In Step 5, because of the excessive number of features/parameters and stimulation conditions, sham subtraction was performed on several selected key features for statistical investigation of the sham-controlled effects induced by each of the two lasers. The statistical analysis utilized for sham-subtracted values was the one-sample t-test, equivalent to paired t-test for sham versus tPBM (gray box in Fig. 3).

Step 1: Quantification of Δ[HbO] and Δ[CCO] time series

Each 7-min data collection consisted of 280 time points (i.e., sampling rate = 0.67 Hz) with an optical spectrum recorded in each time point. The recorded spectrum was in the wavelength range of 740 to 1100 nm. However, previous studies have shown that a spectral band of 780 to 900 nm was sufficient to estimate the chromophores’ concentration efficiently with a relatively low level of error propagated from noise.^52^ The modified Beer-Lambert law was used to estimate Δ[HbO] and Δ[CCO] for each time point,^34^^,^^51^^,^^53^ and corresponding time series was constructed for each of Δ[HbO] and Δ[HbO] under different stimulation conditions and on each lateral side.

Step 2: Spectral analysis of Δ[HbO] and Δ[CCO] time series

To extract the amplitude and phase of each signal, we used the MTM. This method enables to obtain a frequency spectrum with relatively high spectral resolution and low noise using Slepian sequences to taper time series in the time domain followed by the fast Fourier transform.^54^^,^^55^ Specifically, we utilized several functions in MATLAB (including “ft_freqanalysis”) available in the FieldTrip toolbox^56^^,^^57^ to perform MTM operations. The decomposed amplitude and phase in this step were represented as a complex number^58^^,^^59^ that was further used in the connectivity quantification (see Step 3).

Step 3: Hemodynamic and metabolic connectivity/coupling quantification

Connectivity measures, in principle, express the level by which two signals oscillate synchronously. Different methods have been introduced to quantify the amplitude and/or phase of the signals based on the frequency-domain approach.^58^^,^^60^ One of the widely used connectivity measures is coherence, a phase-based frequency-domain analysis that is quantified as a normalized value between 0 and 1. Specifically, the mathematical representation of coherence (COH) for a specific frequency of ω is^58^

$${\mathrm{COH}}_{xy}(\omega )=\frac{\left|S_{xy}(\omega )\right|}{\sqrt{S_{xx}(\omega )S_{yy}(\omega )}},$$ (1)

where $S_{xx}$ and $S_{yy}$ are the power estimates of signals x and y, and $S_{xy}$ is the averaged cross-spectral density of two time series. These terms are calculated using the complex values obtained from the MTM method (see Step 2 above).

In our study, we utilized several functions in MATLAB (including “ft_connectivityanalysis”) available in the FieldTrip toolbox^56^^,^^57^ to perform coherence operations. Each coherence studied in each pair of four signals included: (1) bilateral hemodynamic connectivity between $\Delta [\mathrm{HbO}{]}_{\mathrm{Ipsi}}$ versus $\Delta {\left[\mathrm{HbO}\right]}_{\text{Contra}}$ (${\mathrm{bCON}}_{\text{HbO}}$), (2) bilateral metabolic connectivity between $\Delta {\left[\mathrm{CCO}\right]}_{\mathrm{Ipsi}}$ versus $\Delta [\mathrm{CCO}{]}_{\text{Contra}}$ (${\mathrm{bCON}}_{\text{CCO}}$), (3) unilateral hemodynamic and metabolic coupling on the ipsilateral side between $\Delta {\left[\mathrm{HbO}\right]}_{\mathrm{Ipsi}}$ and $\Delta {\left[\mathrm{CCO}\right]}_{\mathrm{Ipsi}}$ (${\mathrm{uCOP}}_{\text{Ipsi}}$), and (4) unilateral hemodynamic and metabolic coupling on the contralateral side between $\Delta {\left[\mathrm{HbO}\right]}_{\text{Contra}}$ and $\Delta {\left[\mathrm{CCO}\right]}_{\text{Contra}}$ (${\mathrm{uCOP}}_{\text{Contra}}$). The obtained values were then separated into the three ISO frequency bands (E/N/M).

Steps 1 to 3 were repeated for both pre- and post-tPBM stimulations within groups and for both active and sham conditions between groups.

Step 4: Baseline subtraction of coherence

In this study, we quantified the coherence in two 7-min time segments separately, pre- (i.e., baseline) and post-stimulation. For better detection of tPBM/sham effects on coherence, we performed baseline subtraction, rather than baseline normalization, since the baseline coherence in some cases was close to zero. Accordingly, the baseline subtraction for COH indices was expressed as

$$\Delta {\mathrm{COH}}_{i,j}={\mathrm{COH}}_{i,j,\text{post}}-{\mathrm{COH}}_{i,j,\mathrm{pre}},$$ (2)

where i represents the frequency band including endogenic (E), neurogenic (N), and myogenic (M) bands, and j indicates the stimulation condition including R800, R850, RS, L800, and LS. ${\mathrm{COH}}_{i,j,\mathrm{pre}}$ and ${\mathrm{COH}}_{i,j,\text{post}}$ denote the coherence during the 7-min pre- (i.e., baseline) and 7-min post-stimulation period, respectively. Finally, $\Delta {\mathrm{COH}}_{i,j}$, is the baseline-subtracted change in coherence at each of the three ISO frequency bands under different stimulation conditions. This process was repeated for all four coherence indices between bilateral Δ[HbO], bilateral Δ[CCO], and uCOP of Δ[HbO] versus Δ[CCO] on two lateral sides of the prefrontal cortex.

Step 5: Statistical analysis

To account for the sham effect on the changes in the investigated metrics, the $\Delta {\mathrm{COH}}_{i,j}$ values for three tPBM conditions (i.e., R800, L800, and R850) were sham subtracted by subtracting the sham coherence value (i.e., $\Delta {\mathrm{COH}}_{i,\mathrm{sham}}$) from that under tPBM (i.e., $\Delta {\mathrm{COH}}_{i,\mathrm{tPBM}}$)

$$\Delta {\mathrm{COH}}_{i,\mathrm{ss}}=\Delta {\mathrm{COH}}_{i,\mathrm{tPBM}}-\Delta {\mathrm{COH}}_{i,\mathrm{sham}},$$ (3)

As mentioned in Step 4, computed COH can be categorized as either bilateral connectivity (bCON) or uCOP. The sham-subtracted coherence values were then tested with one-sample t-tests for each stimulation condition to identify the sham-controlled significant effects of tPBM in each frequency band separately.

## 3 Results

**Fig. 4:**
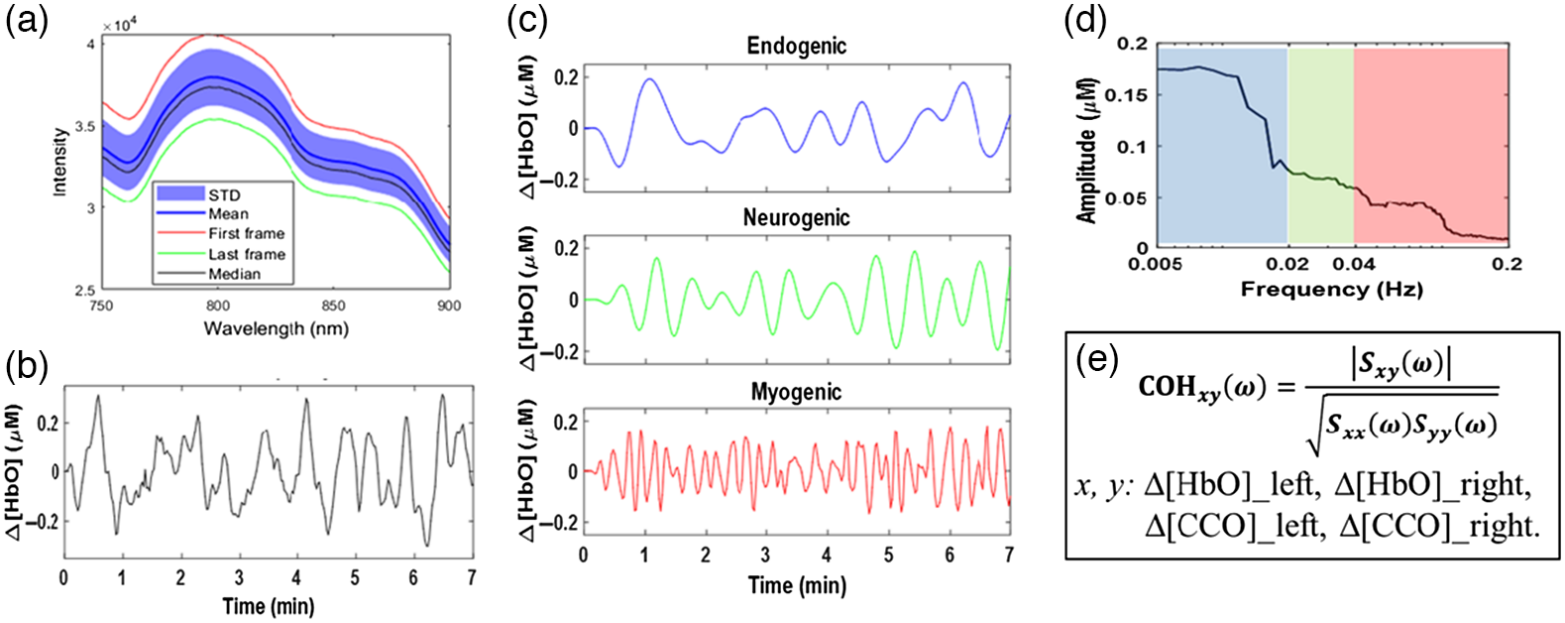
(a) A set of raw optical spectra obtained using bbNIRS averaged over 7 min. This panel shows the mean and median spectra shaded with standard deviation and outlined by the highest and lowest spectra. (b) 7-min time series of Δ[HbO] from a representative participant after converting time-dependent bbNIRS spectra (see the conversion algorithm in the Supplementary Material). (c) Frequency-decomposed time series of the curve in panel (b); it includes three curves for endogenic, neurogenic, and myogenic ISO bands. (d) Quantified spectral amplitude of Δ[HbO] after multi-taper spectral analysis with the endo-, neuro-, and myogenic frequency bands shaded by blue, green, and red boxes, respectively. Finally, all the curves presented in (a)–(d) were calculated repeatedly for Δ[HbO] and Δ[CCO] on each lateral forehead, facilitating (e) coherence quantification among each pair of them.

This study hypothesized that prefrontal tPBM can alter bilateral hemodynamic and metabolic connectivity and unilateral coupling of hemodynamic and metabolic rhythms in the resting human forehead. To support this hypothesis, we performed and analyzed 5 separate experiments, 3 for 8-min active tPBM (i.e., R800, L800, R850) and 2 for 8-min sham stimulation (i.e., RS and LS), from 26 young and healthy participants. Before presenting group-level, baseline-subtracted results for respective quantities, we illustrate in Fig. 4 a demonstrative set of data including (a) group-averaged raw bbNIRS optical spectra, (b) a quantified time series of Δ[HbO]] with the entire ISO frequency range (<0.25  Hz) (c) in three ISO bands, (d) spectral analysis results after the multi-taper method, and (e) a list of quantities for coherence quantification.

To remove baseline variability across participants, coherence-derived connectivity or coupling indices of the ISO of the human forehead were baseline subtracted for both sham and active conditions. For better sensitivity, sham-controlled changes in response to different laser wavelengths and stimulation sites were further quantified and statistically compared for bCON and uCOP, as described in the following two subsections.

### 3.1 tPBM-Induced Alterations in bCON of Forehead Δ[HbO] and Δ[CCO]

**Fig. 5:**
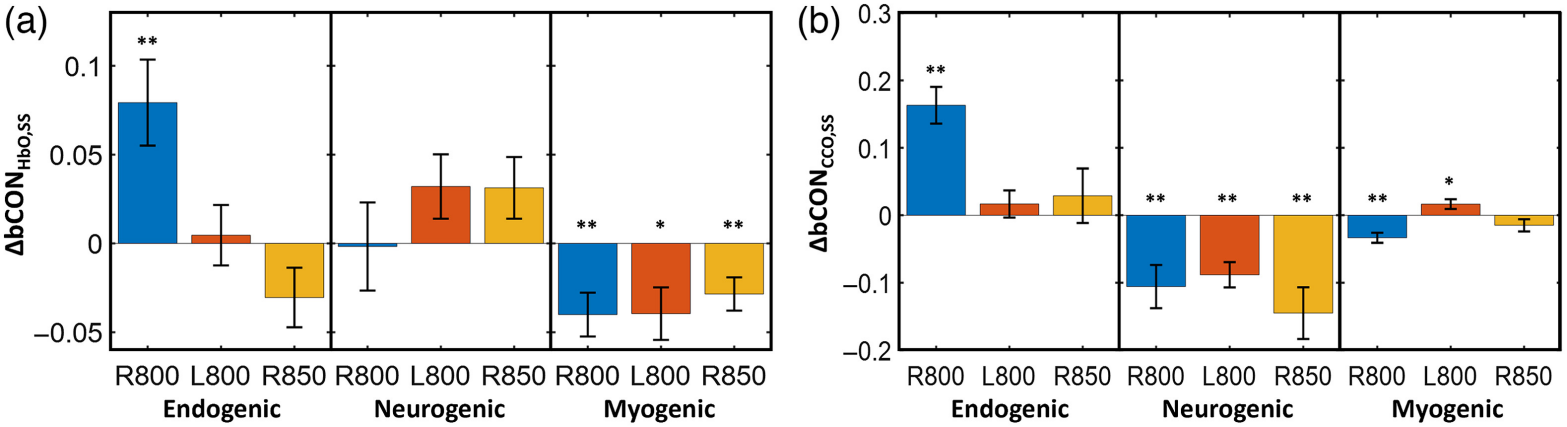
Sham-subtracted tPBM-induced prefrontal (a) $\Delta {\mathrm{bCON}}_{\mathrm{HbO},\mathrm{SS}}$ and (b) $\Delta {\mathrm{bCON}}_{\mathrm{CCO},\mathrm{SS}}$ for different tPBM conditions, namely, R800, L800, R850 at endogenic (E; 0.005 to 0.02 Hz), neurogenic (N; 0.02 to 0.04 Hz), and myogenic (M; 0.04 to 0.2 Hz) frequency bands. Error bars represent the standard error of the mean (n=26). *: p<0.05, **: p<0.01 obtained from one-sample t-tests.

In the case of ${\mathrm{bCON}}_{\mathrm{HbO}}$, all three tPBM conditions induced desynchronization between the two bilateral hemodynamic activities across the human forehead in the myogenic component of ISO, as illustrated in Fig. 5(a). In the case of ${\mathrm{bCON}}_{\mathrm{CCO}}$, all three tPBM conditions led to desynchronization of bilateral metabolism in the neurogenic frequency band [Fig. 5(b)]. In addition, 800-nm tPBM on the right forehead significantly increased both ${\mathrm{bCON}}_{\mathrm{HbO}}$ and ${\mathrm{bCON}}_{\mathrm{CCO}}$ in the endogenic band. Furthermore, the site-specific effect of tPBM on ${\mathrm{bCON}}_{\mathrm{CCO}}$ was noted in the myogenic band, which R800 tPBM resulted in a decrease in bilateral metabolic connectivity, whereas L800 tPBM boosted this connectivity. However, these changes were noticeably much smaller than those in the E and N bands.

### 3.2 tPBM-Induced Alterations in uCOP of Forehead Δ[HbO] and Δ[CCO]

**Fig. 6:**
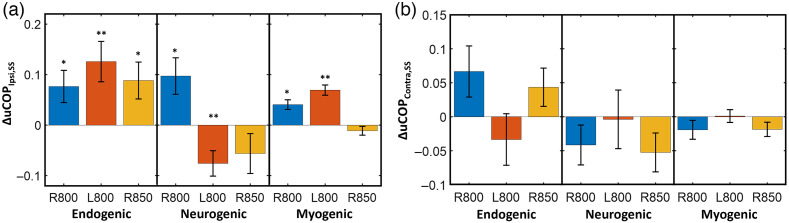
Sham-subtracted tPBM-induced prefrontal (a) $\Delta {\mathrm{uCOP}}_{Ipsi,SS}$ and (b) $\Delta {\mathrm{bCOP}}_{\text{Contra},\mathrm{SS}}$ for different tPBM conditions, namely, R800, L800, R850 at endogenic (E; 0.005 to 0.02 Hz), neurogenic (N; 0.02 to 0.04 Hz), and myogenic (M; 0.04 to 0.2 Hz) frequency bands. Error bars represent the standard error of the mean (n=26). *: p<0.05, **: p<0.01 obtained from one-sample t-tests.

Sham-subtracted unilateral hemodynamic-metabolic coupling on ipsilateral and contralateral sides of the prefrontal cortex (i.e., ${\mathrm{uCOP}}_{\mathrm{Ipsi},\mathrm{SS}}$ and ${\mathrm{uCOP}}_{\text{Contra},\mathrm{SS}}$, respectively) was the other set of metrics examined in this study. As shown in Figs. 6(a) and 6(b), all stimulation conditions significantly affected this set of metrics only in the ipsilateral forehead with respect to the stimulation site; the HbO-CCO coupling over the contralateral side was not modulated significantly. Figure 6(a) reveals three frequency-specific observations: (1) In the endogenic oscillation, the ipsilateral increase in hemodynamic-metabolic coupling was independent of the wavelength and stimulation site. (2) In the myogenic frequency band, the 800-nm laser on either side of the forehead enhanced ${\mathrm{uCOP}}_{\mathrm{Ipsi},\mathrm{SS}}$, whereas this metric was not altered by 850-nm laser illumination on the right forehead. (3) In the neurogenic band, the wavelength- and location-dependent effects of tPBM were more obvious. Right and left 800-nm tPBM resulted in an opposite HbO-CCO coupling effect, whereas the right 850-nm tPBM had no significant effect on the coupling.

## 4 Discussion

NIRS is a widely adopted, low-cost, and noninvasive method for monitoring the hemodynamic and metabolic states of the human brain.^41^^,^^61^^,^^62^ However, it has limitations for neurophysiological characterization of the resting human brain because of the working principle of NIRS,^63^ which permits the determination of only relative changes in each chromophore concentration in the tissue. Our group recently developed a dual-channel bbNIRS system and computational methodology^16^^,^^17^ that enables quantification of resting-state bilateral metabolic and hemodynamic connectivity and unilateral metabolic versus hemodynamic coupling as quantifiable absolute values. In Refs. 16 and 17, we reported the relative stability and reliability of these metrics in healthy young humans.

**Fig. 7:**
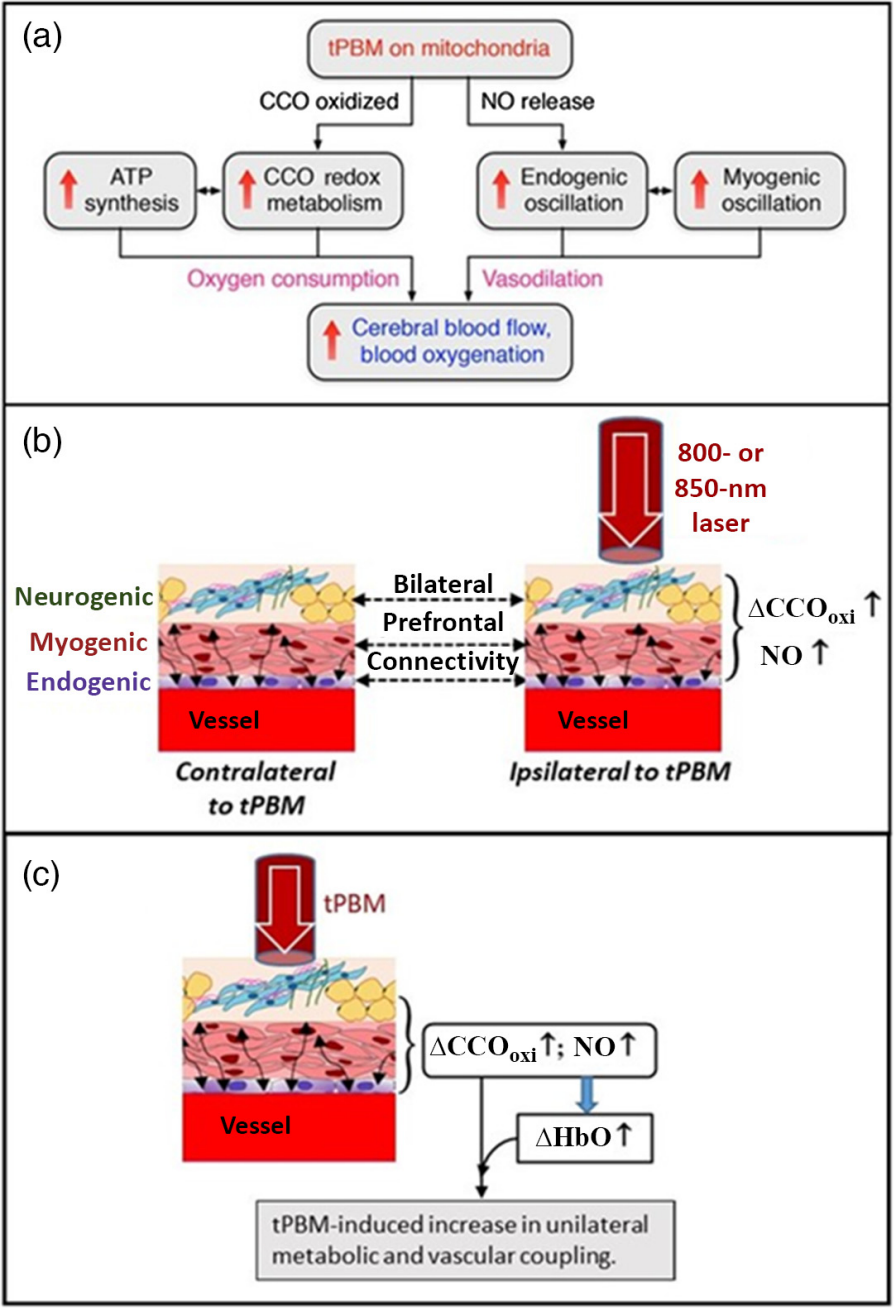
(a) A schematic drawing to show two concurrent metabolic-hemodynamic events occuring within cerebral tissue in response to tPBM.^47^ (b) An illustration to depict bilateral prefrontal connectivity between the ipsilateral and contralateral forehead with respect to the tPBM delivery side. On each lateral side, a piece of blood vessel is surrounded with three anatomical components that facilitate three spontaneous ISO (i.e., endogenic, neurogenic, and myogenic) rhythms, respectively, as labeled on the left side of the panel. This panel demonstrates that either 800- or 850-nm laser enables ipsilateral increases in concentration of oxidized CCO (i.e., $\Delta {\left[\mathrm{CCO}\right]}_{\mathrm{oxi}}$) and NO, both of which alter ISO actions on the contralateral side through bCON at different ISOs. (c) This panel demonstrates uCOP between $\Delta {\left[\mathrm{CCO}\right]}_{\mathrm{oxi}}$ and Δ[HbO] on the stimulation side, indicating that tPBM enhances unilateral metabolic and vascular coupling on the stimulation site and side.

Moreover, in another recent study, we suggested and summarized a mechanistic model with two parallel metabolic-hemodynamic processes induced by tPBM,^47^ as shown in Fig. 7(a). On the one hand, photo-oxidation of CCO stimulates CCO redox metabolism and adenosine triphosphate (ATP) synthesis, leading to a significant increase in Δ[HbO]. On the other hand, tPBM triggers the release of nitric oxide (NO),^64^^,^^65^ thus increasing changes in endogenic and myogenic oscillations. Consequently, NO-derived vasodilation causes an increase in cerebral blood flow. However, this mechanistic interpretation did not include any frequency-specific prefrontal connectivity or unilateral metabolic-hemodynamic coupling in response to tPBM. In this study, we focused on the site- and frequency-specific effects of forehead tPBM with 800- and 850-nm lasers on neurophysiological networks in the resting human brain, including bilateral hemodynamic/metabolic connectivity and unilateral hemodynamic-metabolic coupling.

### 4.1 Site- and Frequency-Specific Effects of tPBM on bCON

bCON represents the coherence of oscillations in the concentration of a specific chromophore (Δ[HbO] or Δ[CCO]) over the lateral prefrontal cortices. This functional connectivity reflects whether cerebral (hemodynamic or metabolic) activity functions bilaterally or locally.^66^ Moreover, a change in this metric in response to local neural stimulation indicates whether this perturbation can neuromodulate the human brain remotely or only near the stimulation site.

As illustrated in Fig. 5(a), all three stimulation conditions induced desynchronization/reduction of bilateral hemodynamic connectivity in the myogenic band. This observation is in good agreement with the results reported using 1064-nm tPBM,^16^ indicating that prefrontal tPBM enables local neuromodulation of smooth muscle cells in the ipsilateral vasculature with respect to the light delivery side. The tPBM-induced myogenic reduction in bCON implies that light stimulation caused myogenic oscillations out of synchronization with respect to those on the contralateral side. It is reasonable to speculate that the bCON through the prefrontal smooth muscles of the blood vessels was still active, but with a delayed alteration motion, causing a reduction in connectivity (i.e., ${\mathrm{bCON}}_{\mathrm{HbO},\mathrm{ss}}$). Furthermore, this modulation of myogenic connectivity was not site- or wavelength-specific for the tPBM. In addition, the same tPBM-induced desynchronization occurred in the neurogenic band of ${\mathrm{bCON}}_{\mathrm{CCO},\mathrm{SS}}$. This desynchronization is expected to result from the local photo-oxidation of CCO within the mitochondria,^67^^–^^69^ especially in the axon terminals of cortical neurons near the stimulation site, where the release of neurotransmitters modulates the oscillation in the neurogenic band. Following a similar interpretation to that of ${\mathrm{bCON}}_{\mathrm{HbO},\mathrm{ss}}$, we speculated that the bilateral mitochondrial connection was active through neurogenic oscillation but with a delayed alteration rate on the contralateral side, causing a reduction in connectivity in ${\mathrm{bCON}}_{\mathrm{CCO},\mathrm{ss}}$. Figure 7(b) illustrates the bCON at three specific ISO frequencies in response to forehead tPBM for the results shown in Fig. 5.

Furthermore, the right-forehead tPBM by 800-nm laser significantly enhanced bilateral hemodynamic (${\mathrm{bCON}}_{\mathrm{HbO},\mathrm{SS}}$) and metabolic (${\mathrm{bCON}}_{\mathrm{CCO},\mathrm{SS}}$) connectivity only in the endogenic band, as clearly seen in Figs. 5(a) and 5(b). This observation highlights that the right prefrontal 800-nm tPBM has the ability to remotely neuromodulate the endothelial oscillation of vascular and metabolic activity on the contralateral side to the stimulation site. It also implies that the bilateral hemodynamic and mitochondrial association/link was highly active through endogenic oscillation without any delay on the contralateral side, leading to a significant increase in bilateral coherence in both ${\mathrm{bCON}}_{\mathrm{HbO},\mathrm{SS}}$ and ${\mathrm{bCON}}_{\mathrm{CCO},\mathrm{ss}}$.

Another demonstration of the location- and frequency-related effect of tPBM is the alteration of ${\mathrm{bCON}}_{\mathrm{CCO}}$ in the myogenic band, where the right and left 800-nm lasers resulted in opposite modulation effects of bilateral metabolic connectivity [Fig. 5(b)]. In other words, the right 800-nm laser desynchronized the smooth muscle-sourced oscillations of Δ[CCO] bilaterally and perturbed it toward more locally driven (more segregated) activity. In contrast, the left 800-nm laser boosted the integration of bilateral metabolic activities over the prefrontal regions. The interpretation of this observation is beyond the current knowledge and requires further investigation.

### 4.2 Site- and Frequency-Specific Effect of tPBM on uCOP

uCOP between hemodynamic and metabolic activities in the same lateral forehead or prefrontal cortex denotes how oxygen demand (represented by Δ[CCO]) and supply (represented by Δ[HbO]) are regulated in that specific cerebral region. Previous studies by our group have revealed a stable level of this metric in young healthy adults.^17^ However, irregularities in the cerebral supply-demand relationship exist in a variety of neurological impairments or disorders.^70^^–^^72^

As reported in Sec. 3.2, unilateral hemodynamic-metabolic coupling was significantly photobiomodulated only on the ipsilateral side [Fig. 6(a)] relative to the stimulation site/side. No significant changes were observed on the contralateral side of the prefrontal cortex [Fig. 6(b)]. Specifically, ${\mathrm{uCOP}}_{\mathrm{Ipsi}}$ had a significant increase in the endogenic ISO in response to all three stimulation conditions. This observation implies an enhanced and more robust oxygen demand versus supply relationship in endothelial oscillations mediated by the mitochondrial ATP synthesis cycle, which is boosted by tPBM-induced photooxidation of CCO. Accordingly, as illustrated in Figs. 7(a) and 7(c), tPBM facilitates increases in both oxidized CCO ($\Delta {\left[\mathrm{CCO}\right]}_{\mathrm{oxi}}$) and NO, both of which stimulate the endothelial layers of the local cerebral vasculature, thus promptly enhancing unilateral metabolic and vascular coupling on the stimulation site and side.

### 4.3 Wavelength-Specific Effect of tPBM on bCON and uCOP

The effect of laser wavelength is noticeable in the bCON of $\Delta {\mathrm{bCON}}_{\mathrm{HbO},\mathrm{SS}}$ and $\Delta {\mathrm{bCON}}_{\mathrm{CCO},\mathrm{SS}}$ in the endogenic band. Figure 5 reveals a significant enhancement of $\Delta {\mathrm{bCON}}_{\mathrm{HbO},\mathrm{SS}}$ and $\Delta {\mathrm{bCON}}_{\mathrm{CCO},\mathrm{SS}}$ by right 800-nm tPBM, but the right 850-nm laser did not create any significant alteration in either of these endogenic connectivities. Similarly, the right 850-nm tPBM did not perturb bilateral metabolic connectivity ($\Delta {\mathrm{bCON}}_{\mathrm{CCO},\mathrm{SS}}$) in the myogenic frequency band [Fig. 5(b)]. Moreover, regarding uCOP, while the right 800-nm tPBM significantly altered ${\mathrm{uCOP}}_{\mathrm{Ipsi},\mathrm{SS}}$ in all three ISO rhythm bands, the 850-nm laser did not significantly perturb vascular-metabolic coupling in neurogenic and myogenic oscillations [Fig. 6(a)]. In short, 800-nm prefrontal tPBM seemed to be more significant and effective than 850-nm to modulate both bCON and uCOP.

Wavelength-specific effects may be attributed to differences in light scattering and absorption in the cerebral vasculature. It is known that light scattering of cerebral tissue at 800 nm is slightly higher than that at 850 nm.^73^^,^^74^ Thus, 800-nm light would cover a slightly larger cortical area caused by more light scattering. In contrast, 800-nm light is absorbed more by the cerebral vasculature than the 850-nm laser, so more optical irradiance at 800 nm would be deposited in the cortical region and lead to higher light stimulation,^74^ bCON, and uCOP. Such expectation or explanation needs to be further confirmed in future studies.

### 4.4 Applications of Different Stimulation Setting Conditions of tPBM

In Secs. 4.1 and 4.2, we established that prefrontal tPBM to the resting human forehead enabled significant alterations in bCON and uCOP of cerebral neurophysiology in the three ISO rhythms. However, these alterations are wavelength- and site-specific in the resting human brain. These findings are in excellent agreement with a recent publication that reported significant enhancements of visual working memory capacity in humans by tPBM.^75^ In particular, through four experiments using two separate laser wavelengths (850 nm and 1064 nm) and two stimulation sites (left and right forehead), Zhao et al. showed that effectiveness of tPBM will depend on stimulation parameters, such as the power density, wavelength, dosage, and location.^75^

Studies have shown that each ISO frequency component is associated with a specific neurophysiological activity in the healthy human brain.^22^^–^^24^^,^^76^ Therefore, impaired or diminished infra-slow activity in cerebral hemodynamic and metabolic functions may serve as a potential indicator of neurological or metabolic disorders. For example, several studies have shown the relation between the impairment of infra-slow cerebral activity and cardiovascular disease, Alzheimer’s disease, hypertension, and stroke.^14^^,^^15^^,^^77^ In addition, the prefrontal cortex has been shown to be closely associated with cognitive function; thus, many cognitive impairments or disorders (e.g., Alzheimer’s disease) are also caused by neurological malfunction in this cortical region.^78^^–^^82^

As suggested by our previous studies,^16^^,^^17^ the proposed bCON and uCOP metrics of the resting human prefrontal cortex are stable with relatively high reproducibility among young, healthy humans. These resting-state, frequency-specific metrics may serve as potential features for identifying neurophysiological disorders because such frequency bands are highly associated with neurophysiological activities in the human brain. Furthermore, as discussed above, these metrics may help better understand the mechanism of action behind different stimulation conditions of tPBM. However, this study clearly demonstrates that these metrics need to be further investigated before tPBM can become an effective intervention tool. A crucial step toward this goal is to associate or correlate tPBM-induced alterations of quantified bCON and uCOP with tPBM-induced behavioral improvement in the studied neurological impairment or disorder.^83^^–^^86^

### 4.5 Limitations and Future Work

First, some participants may have been drowsy during the data acquisition period because of the eyes-closed measurement requirement. Based on the operator’s observations and self-reports from the participants, data from excessively sleepy subjects were excluded as outliers. However, the eyes-closed resting state may have caused sleepiness in all subjects, especially during the post-stimulation period, causing some unintentional head movements. Because bbNIRS is usually sensitive to motion artifacts and measures cerebral metabolic/hemodynamic activities, sleepiness may add unwanted physical and neurophysiological noise induced by both head motions and sleepiness. Second, our quantified metrics may be potentially contaminated by extracranial layers of the human head, namely, the scalp and skull. Extra channels with shorter source–detector (S-D) separation have been used in task-evoked hemodynamic studies^87^^–^^91^ to minimize this potential confounding factor. However, few applicable methodologies have been developed to remove this contaminating noise in multi-channel fNIRS-based resting-state studies until a recent report,^92^ which demonstrated a solution by utilizing a multi-channel setup along with principle component analysis. Finally, this study investigated the effects of tPBM with limited (800 and 850 nm) wavelengths on one lateral (right) forehead and only one (800 nm) wavelength on both lateral foreheads. The conclusions given in the study need to be further confirmed as a generalized finding.

As for future work, advances of the 2-bbNIRS system are needed to minimize the head movement, sleepiness effects, and contaminations from extracranial layers. Also, to be able to select an optimal wavelength and stimulation site for tPBM intervention, concurrent measurements and correlations between behavioral (or neuropsychological) and neurophysiological alterations induced by tPBM would be beneficial for a specific neurological application.

## 5 Conclusion

In general, this study supported our hypothesis that hemodynamic and metabolic activities in the ISO of the resting human forehead are significantly modulated by tPBM stimulation conditions and that the modulation is wavelength- and site-specific, as well as distinct in different ISO bands. Specifically, the 7-min, dual-channel bbNIRS measurements before and after 8-min of tPBM combined with coherence analysis enabled us to identify consistent alterations induced by prefrontal tPBM at 800 and 850 nm on the human forehead. These alterations included (1) desynchronization of bilateral hemodynamic activity in the myogenic band, (2) decrease in bilateral metabolic connectivity in the neurogenic band, and (3) increase in unilateral hemodynamic-metabolic coupling in the endogenic band on the ipsilateral side of the stimulation. On the one hand, the reported observations reaffirm the local effect of tPBM on neurophysiological activities. On the other hand, we demonstrated that the neurophysiological networks or effects altered by prefrontal tPBM are wavelength- and site-specific in the resting human brain. This phenomenon is specifically observed in the bilateral effects of the right prefrontal 800-nm stimulation on endothelial-mediated hemodynamic and metabolic oscillations. For future studies that wish to apply tPBM to enhance cognitive function or treat neurophysiological disorders, a more detailed investigation on the selection of tPBM setting conditions is needed for effective behavioural and neurophysiological improvement.

## Code, Data, and Materials Availability

The research code, data, and materials supporting this publication are available upon request from the corresponding author.
