# Supplementary material for: Site-specific effects of 800- and 850-nm forehead transcranial photobiomodulation on prefrontal bilateral connectivity and unilateral coupling in young adults
Source: Neurophotonics. 2023 Jun 5;10(2):025012. doi: 10.1117/1.NPh.10.2.025012 (PMC10240350; doi:10.1117/1.NPh.10.2.025012)
Supplement: Supplementary file 1 [file NPh_010_025012_SD001.pdf]

Site-specific effects of 800- and 850-nm forehead transcranial photobiomodulation on prefrontal bilateral connectivity and unilateral coupling in young adults

Sadra Shahdadian, Xinlong Wang, Shu Kang, Caroline Carter, and Hanli Liu*
Department of Bioengineering, University of Texas at Arlington, 500 UTA Blvd, Arlington, TX 76019, USA

Supplementary Material

Theoretical Foundation for Data Processing

Methods to quantify changes in concentrations of oxygenated hemoglobin (Δ[HbO]), deoxygenated hemoglobin (Δ[HHb]), and redox-state cytochrome c oxidase (Δ[CCO]) have been developed and reported [1, 2]. A brief review is provided below for general readers who wish to understand the theoretical foundation and processing methods in depth.

A broadband near-infrared spectroscopy (bbNIRS) system provides measurements of optical spectra at different times (t), as expressed *I(t, λ)*. A relative optical density spectrum, *ΔOD*(*t*, *λ*), can be defined and calculated at each wavelength λ as:

$$\Delta OD(t,\lambda) = log_{10}[\frac{I_0(t=0,\lambda)}{I(t,\lambda)}], \quad (1)$$

where $I_0(t=0, \lambda)$ can be the baseline spectrum at time *t*=0 or an average of several initial baseline spectral readings (i.e., the first two spectra collected in each experiment), and *I(t, λ)* represent time-varying spectra acquired at each time point throughout the entire experiment. The estimations of Δ[HbO] and Δ[CCO] from raw spectral data taken with bbNIRS throughout the experiment were based on modified Beer-Lambert's law [3], which offers a quantitative relationship of *ΔOD(λ)* on *Δ[HbO], Δ[HHb], and Δ[CCO]* at each wavelength, *λ*, at each time point, with a wavelength-dependent path-length factor, *L(λ)*. Based on optical diffusion theory [4], *ΔOD(λ)*/*L(λ)* can be expressed as a sum of optical absorbance contributed by *Δ[HbO]*, *Δ[HHb]*, and *Δ[CCO]* components, as given below:

$$\begin{bmatrix} \frac{\Delta OD(\lambda_1)}{L(\lambda_1)} \\ \frac{\Delta OD(\lambda_2)}{L(\lambda_2)} \\ \frac{\Delta OD(\lambda_3)}{L(\lambda_3)} \\ \ldots \\ \frac{\Delta OD(\lambda_n)}{L(\lambda_n)} \end{bmatrix} = \Delta[HbO]*\begin{bmatrix} \varepsilon_{HbO}(\lambda_1) \\ \varepsilon_{HbO}(\lambda_2) \\ \varepsilon_{HbO}(\lambda_3) \\ \ldots \\ \varepsilon_{HbO}(\lambda_n) \end{bmatrix} + \Delta[HHb]*\begin{bmatrix} \varepsilon_{HHb}(\lambda_1) \\ \varepsilon_{HHb}(\lambda_2) \\ \varepsilon_{HHb}(\lambda_3) \\ \ldots \\ \varepsilon_{HHb}(\lambda_n) \end{bmatrix} + \Delta[CCO]*\begin{bmatrix} \varepsilon_{CCO}(\lambda_1) \\ \varepsilon_{CCO}(\lambda_2) \\ \varepsilon_{CCO}(\lambda_3) \\ \ldots \\ \varepsilon_{CCO}(\lambda_n) \end{bmatrix}, \quad (2)$$

where *Δ[HbO], Δ[HHb] and Δ[CCO]* are relative concentration changes of HbO, HHb and CCO respectively; $\varepsilon_{HbO}(\lambda)$, $\varepsilon_{HHb}(\lambda)$ and $\varepsilon_{CCO}(\lambda)$ represent the extinction coefficients at each wavelength of HbO, HHb and CCO, which can be found in ref. [1]; *L(λ)* is a wavelength dependent factor that denotes the effective pathlength of the detected photons through tissues at each wavelength. Furthermore, according to the Modified Beer-Lambert Law [3, 5], *L(λ)* can be expressed as:

$$\begin{bmatrix} L(\lambda_1) \\ L(\lambda_2) \\ L(\lambda_3) \\ \ldots \\ L(\lambda_n) \end{bmatrix} = r*\begin{bmatrix} DPF(\lambda_1) \\ DPF(\lambda_2) \\ DPF(\lambda_3) \\ \ldots \\ DPF(\lambda_n) \end{bmatrix}, \quad (3)$$

where *r* is a constant that denotes the source-detector distance. In this study, we used source detector separation of 3 cm, so *r*=3. The wavelength dependence of *L(λ)* is caused by a wavelength-dependent differential pathlength factor, *DPF(λ)*. By substituting Eq. (3) into Eq. (2) for multiple wavelengths, the estimation of *Δ[HbO], Δ[HHb] and Δ[CCO]* can be expressed as follows:

$$\begin{bmatrix} \Delta[HbO] \\ \Delta[HHb] \\ \Delta[CCO] \end{bmatrix} = \frac{1}{r}*\begin{bmatrix} \varepsilon_{HbO}(\lambda_1) & \varepsilon_{HHb}(\lambda_1) & \varepsilon_{CCO}(\lambda_1) \\ \varepsilon_{HbO}(\lambda_2) & \varepsilon_{HHb}(\lambda_2) & \varepsilon_{CCO}(\lambda_2) \\ \ldots & & \\ \varepsilon_{HbO}(\lambda_n) & \varepsilon_{HHb}(\lambda_n) & \varepsilon_{CCO}(\lambda_n) \end{bmatrix}^{-1} \begin{bmatrix} \frac{\Delta OD(\lambda_1)}{DPF(\lambda_1)} \\ \frac{\Delta OD(\lambda_2)}{DPF(\lambda_2)} \\ \ldots \\ \frac{\Delta OD(\lambda_n)}{DPF(\lambda_n)} \end{bmatrix}. \quad (4)$$

In order to accurately solve *Δ[HbO], Δ[HHb] and Δ[CCO]* using Eq. (4), we would need to know $DPF(\lambda)$ in the wavelength range of our measurements. It is known that appropriate or accurate selection/estimation of wavelength-dependent DPF is crucial for accurate estimation of chromophore concentrations [6]. In this study, DPF(λ) values were assumed to be time-invariant because of given stable brain optical properties. Based on diffusion theory with the semi-infinite boundary geometry [7], $DPF(\lambda)$ can be determined by

$$DPF(\lambda)=\frac{\sqrt{3\mu_s'(\lambda)}}{2\sqrt{\mu_a(\lambda)}}*\frac{r\sqrt{3\mu_a(\lambda)\mu_s'(\lambda)}}{r\sqrt{3\mu_a(\lambda)\mu_s'(\lambda)}+1} \tag{5}$$

where $\mu_a(\lambda)$ and $\mu_s'(\lambda)$ are the estimated absorption and reduced scattering coefficients across the wavelength range of interest.

Values of $\mu_a(\lambda)$ and $\mu_s'(\lambda)$ were measured using a tissue oximeter (OxiplexTS, ISS) that operates in the frequency-domain. This device provides readings of μ_a and μ_s' values at 750 nm and 830 nm, as well as absolute concentrations of [HbO] and [HHb]. However, to obtain $\mu_s'(\lambda)$ values across the entire range of wavelengths from 780-900 nm, we used Mie theory to interpolate and extrapolate the two measured μ_s' values at 750 nm and 830 nm. Mie theory is typically represented by $k\lambda^{-b}$, where k and b were determined by fitting this equation to both μ_s' values at 750 nm and 830 nm. In addition, absorption coefficients in the same wavelength range (780-900 nm) were estimated based on [HbO] and [HHb] measured by the same tissue oximeter [4].

After combining the measured $\Delta OD(\lambda)$ values across the measurement period and empirical $\mu a(\lambda)$ and $\mu s'(\lambda)$ values of the human forehead [2], we were able to solve eq. (4) at each measurement time point using MATLAB, achieving temporal series of *Δ[HbO], Δ[HHb]* and *Δ[CCO]* under respective experimental conditions, as shown in Fig. 4(b) in the main paper. Specifically, our calculations covered the spectral range of 780-900 nm with a total of 121 wavelengths. Figure S1 below illustrates the processing steps described above.

Step 1: bbNIRS data acquisition to form time-dependent optical spectra in the NIR range of 750-900 nm.

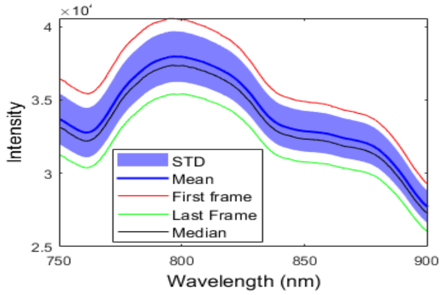

Step 2: Calculation of ΔOD spectra at each time point. t.

$$\Delta OD(t,\lambda) = log_{10}[\frac{I_0(t=0,\ \lambda)}{I(t,\lambda)}]$$

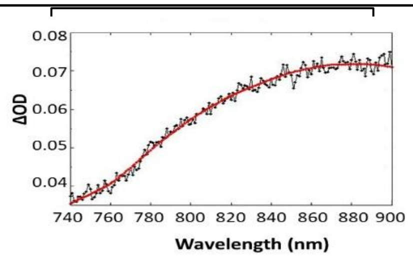

Step 3: Modified Beer-Lambert's law that associates measured ΔOD values over *n* wavelengths with changes of concentrations in *Δ[HbO]*, *Δ[HHb]* and *Δ[CCO]*.

$$\begin{cases} \Delta OD(\lambda_1) = r * DPF_{t0}(\lambda_1) * \{\Delta[HbO] * \varepsilon_{HbO}(\lambda_1) + \Delta[HHb] * \varepsilon_{Hb}(\lambda_1) + \Delta[CCO] * \varepsilon_{CCO}(\lambda_1)\} \\ \Delta OD(\lambda_2) = r * DPF_{t0}(\lambda_2) * \{\Delta[HbO] * \varepsilon_{HbO}(\lambda_2) + \Delta[HHb] * \varepsilon_{Hb}(\lambda_2) + \Delta[CCO] * \varepsilon_{CCO}(\lambda_2)\} \\ \Delta OD(\lambda_3) = r * DPF_{t0}(\lambda_3) * \{\Delta[HbO] * \varepsilon_{HbO}(\lambda_3) + \Delta[HHb] * \varepsilon_{Hb}(\lambda_3) + \Delta[CCO] * \varepsilon_{CCO}(\lambda_3)\} \\ \vdots \\ \Delta OD(\lambda_n) = r * DPF_{t0}(\lambda_n) * \{\Delta[HbO] * \varepsilon_{HbO}(\lambda_n) + \Delta[HHb] * \varepsilon_{Hb}(\lambda_n) + \Delta[CCO] * \varepsilon_{CCO}(\lambda_n)\} \end{cases}$$

Step 4: Quantification of *Δ[HbO]* and *Δ[CCO]* by solving the following matrix at each time point after performing the pseudo-inversion of the *n*×3 ε matrix. Then, a time-dependent series of *Δ[HbO]* (and *Δ[CCO]*) can be formed, as demonstrated below on the right panel.

$$\begin{bmatrix} \Delta[HbO] \\ \Delta[HHb] \\ \Delta[CCO] \end{bmatrix} = \frac{1}{r} * \begin{bmatrix} \varepsilon_{HbO}(\lambda_1) & \varepsilon_{HHb}(\lambda_1) & \varepsilon_{CCO}(\lambda_1) \\ \varepsilon_{HbO}(\lambda_2) & \varepsilon_{HHb}(\lambda_2) & \varepsilon_{CCO}(\lambda_2) \\ \cdots & & \\ \varepsilon_{HbO}(\lambda_n) & \varepsilon_{HHb}(\lambda_n) & \varepsilon_{CCO}(\lambda_n) \end{bmatrix}^{-1} \begin{bmatrix} \frac{\Delta OD(\lambda_1)}{DPF(\lambda_1)} \\ \frac{\Delta OD(\lambda_2)}{DPF(\lambda_2)} \\ \cdots \\ \frac{\Delta OD(\lambda_n)}{DPF(\lambda_n)} \end{bmatrix}$$

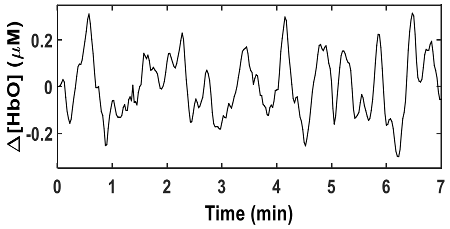

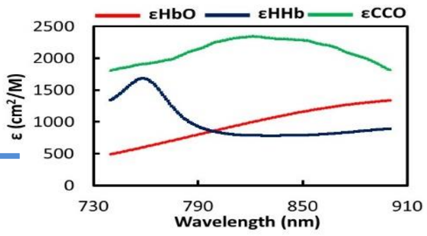

Fig. S1 A data processing flow chart used to quantify *Δ[HbO]* and *Δ[HHb]* from raw bbNIRS data.

References:

[1] C. Kolyva *et al.*, "Systematic investigation of changes in oxidized cerebral cytochrome c oxidase concentration during frontal lobe activation in healthy adults," *Biomed Opt Express*, vol. 3, no. 10, pp. 2550-66, Oct 1 2012, doi: 10.1364/BOE.3.002550.

[2] X. Wang *et al.*, "Up-regulation of cerebral cytochrome-c-oxidase and hemodynamics by transcranial infrared laser stimulation: A broadband near-infrared spectroscopy study," *J Cereb Blood Flow Metab*, vol. 37, no. 12, pp. 3789-3802, Dec 2017, doi: 10.1177/0271678X17691783.

[3] L. Kocsis, P. Herman, and A. Eke, "The modified Beer-Lambert law revisited," *Phys Med Biol,* vol. 51, no. 5, pp. N91-8, Mar 7 2006, doi: 10.1088/0031-9155/51/5/N02.

[4] X. Wang, F. Tian, S. S. Soni, F. Gonzalez-Lima, and H. Liu, "Interplay between up-regulation of cytochrome-c-oxidase and hemoglobin oxygenation induced by near-infrared laser," *Sci Rep,* vol. 6, p. 30540, 2016, doi: 10.1038/srep30540.

[5] F. Scholkmann *et al.*, "A review on continuous wave functional near-infrared spectroscopy and imaging instrumentation and methodology," *NeuroImage*, vol. 85 Pt 1, pp. 6-27, Jan 15 2014, doi: 10.1016/j.neuroimage.2013.05.004.

[6] S. J. Matcher, M. Cope, and D. T. Delpy, "Use of the water absorption spectrum to quantify tissue chromophore concentration changes in near-infrared spectroscopy," *Phys Med Biol,* vol. 39, no. 1, pp. 177-96, Jan 1994. [Online]. Available: https://www.ncbi.nlm.nih.gov/pubmed/7651995.

[7] S. Fantini *et al.*, "Non-invasive optical monitoring of the newborn piglet brain using continuous-wave and frequency-domain spectroscopy," *Phys Med Biol*, vol. 44, no. 6, pp. 1543-63, Jun 1999. [Online]. Available: http://www.ncbi.nlm.nih.gov/pubmed/10498522.
